# Innate immune mediator, Interleukin-1 receptor accessory protein (IL1RAP), is expressed and pro-tumorigenic in pancreatic cancer

**DOI:** 10.1186/s13045-022-01286-4

**Published:** 2022-05-23

**Authors:** Yang Zhang, Xiaoyi Chen, Huamin Wang, Shanisha Gordon-Mitchell, Srabani Sahu, Tushar D. Bhagat, Gaurav Choudhary, Srinivas Aluri, Kith Pradhan, Plabani Sahu, Milagros Carbajal, Hui Zhang, Beamon Agarwal, Aditi Shastri, Robert Martell, Daniel Starczynowski, Ulrich Steidl, Anirban Maitra, Amit Verma

**Affiliations:** 1 Albert Einstein College of Medicine, Montefiore Medical Center, 1300 Morris Park Avenue, Bronx, NY 10461 USA; 2 Departments of Anatomical Pathology, University of Texas MD Anderson Cancer Center, Houston, TX USA; 3 Translational Molecular Pathology, University of Texas MD Anderson Cancer Center, Houston, TX USA; 4 GenomeRxUs LLC, Secane, PA USA; 5 Curis Inc, Lexington, MA USA; 6 Cincinnati Children’s Hospital, Cincinnati, OH USA

**Keywords:** Pancreatic cancer, IL1RAP, IRAK4

## Abstract

Advanced pancreatic ductal adenocarcinoma (PDAC) is usually an incurable malignancy that needs newer therapeutic targets. Interleukin-1 receptor accessory protein (IL1RAP) is an innate immune mediator that regulates activation of pro-inflammatory and mitogenic signaling pathways. Immunohistochemistry on tissue microarrays demonstrated expression of IL1RAP in majority of human PDAC specimens and in murine pancreatic tumors from K-Ras^G122D^/p53^R172H^/PDXCre (KPC) mice. Single cell RNA-Seq analysis of human primary pre-neoplastic lesions and adenocarcinoma specimens indicated that overexpression occurs during carcinogenesis. IL1RAP overexpression was associated with worse overall survival. IL1RAP knockdown significantly reduced cell viability, invasiveness, and clonogenic growth in pancreatic cancer cell lines. Inhibition of the downstream interleukin-1 receptor-associated kinase 4 (IRAK4) using two pharmacologic inhibitors, CA-4948 and PF06650833, resulted in reduced growth in pancreatic cancer cell lines and in xenograft models.

**Supplementary Information:**

The online version contains supplementary material available at 10.1186/s13045-022-01286-4.

**To the editor,**

Pancreatic ductal adenocarcinoma (PDAC) is a lethal malignancy that needs newer therapeutic targets. The surface molecule, interleukin-1 receptor accessory protein (IL1RAP), is consistently overexpressed across multiple genetic subtypes of acute myeloid leukemia (AML) and chromic myeloid leukemia, including at the stem cell level [1, 2]. Since IL1RAP has pro-proliferative roles in cancer models and is involved in potentiating cytokine signaling, we examined its functional roles in pancreatic cancer.

To determine the expression of IL1RAP in human primary PDAC samples, immunohistochemical staining was done on human PDAC tissue microarrays. We observed that most PDAC samples (212/262, 81%) showed 1 + to 3 + staining for IL1RAP in tumor cells (Fig. 1A,B). Next, we evaluated whether increased IL1RAP expression was observed in PDAC samples from the KPC (K-Ras^G122D^/p53^R172H^/PDXCre) [3] mouse model. Immunostaining demonstrated that the PDAC tumors from KPC mice robustly expressed the IL1RAP protein when compared to pre-neoplastic lesions as well as normal pancreatic samples obtained from wild type mice (Fig. 1C–E). To validate IL1RAP expression at single cell levels, we next analyzed previously published dataset of single cell RNA-seq data from samples obtained from precancerous low grade intraductal papillary mucinous neoplasm (IPMN), high grade IPMN and PDAC associated with IPMN [4] (Fig. 1F–H). IL1RAP expression was seen in EpCAM positive neoplastic cells and was increased in PDAC associated with IPMN. These data demonstrated IL1RAP was overexpressed at both mRNA and protein levels in PDACs. Next, we determine the prognostic value of IL1RAP expression in human PDAC samples (TCGA, *N* = 179) and observed that higher expression of IL1RAP was associated with worse overall survival (Fig. 1I, high and low expression based on median, KM curve with log rank *p val* = 0.002).

**Fig. 1:**
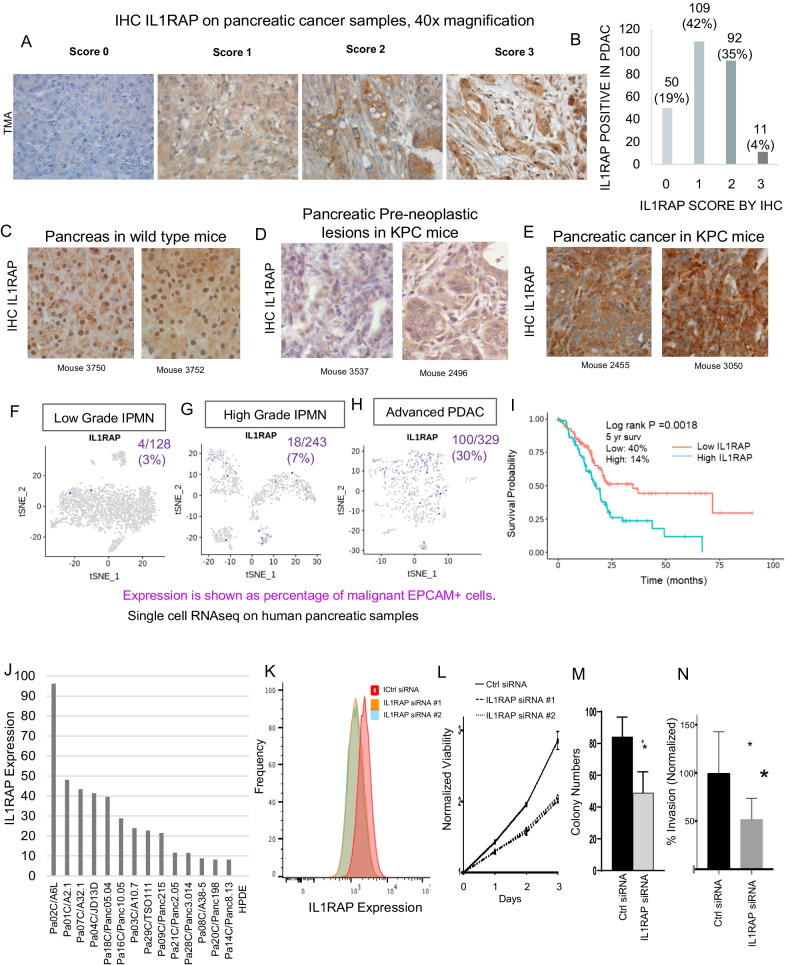
IL1RAP is overexpressed in pancreatic cancer. **A**, **B**: Tissue microarrays containing human pancreatic ductal adenocarcinoma (PDAC) samples were stained with antibodies against IL1RAP and examined by immunohistochemistry. Representative scores for both TMAs are shown. Majority (81%) of PDAC samples demonstrated positivity for IL1RAP expression. **C**–**E**: Tissues from wildtype mouse pancreas (**C**), preneoplastic lesions (**D**) and pancreatic cancer (**E**) in KRAS mutant, TP53 mutant (KPC) mouse model were obtained and stained with antibody against IL1RAP. Increased membranous intensity of IL1RAP expression was seen in PDAC samples from KPC mouse. **F**–**H**: Single cell RNAseq done on samples from human pre-neoplastic lesions (IPMN, F), High risk IPMN (**G**) and advanced PDAC (**H**) tumors was examined for IL1RAP expression (shown in purple). The percentage of IL1RAP positive cells is expressed per total EPCAM positive cells. **I**: Analysis of 176 PDAC samples (TCGA) shows that higher expression of IL1RAP is associated with worse overall survival. High and low expression based on median, KM curve with log rank *P* value = 0.012. **J**: IL1RAP expression in PDAC derived cell lines and ductal control (HPDE) shows increased expression in A6L and other cell lines. **K**: siRNA mediated reduction in IL1RAP expression is seen in A6L PDAC cells by flow cytometry. **L**: siRNA mediated knockdown of IL1RAP leads to reduced viability in A6L PDAC cells. Means ± stdev, *N* = 8. Ttest *P* Val < 0.01. **M**: siRNA mediated knockdown of IL1RAP leads to reduced colony growth in A6L PDAC cells. Means ± stdev, *N* = 10 *P* Val < 0.01. **N**: siRNA mediated knockdown of IL1RAP leads to reduced invasiveness in A6L PDAC cells. Means ± stdev, *N* = 12. Ttest *P* Val < 0.01

We also evaluated IL1RAP expression in a large panel of PDAC cell lines [5] and compared to human pancreatic ductal control (HPDE) cells. All 14 PDAC cell lines examined expressed higher IL1RAP than HPDE cells with the A6L (Pa02C) cell line showing the highest expression of IL1RAP (Fig. 1J). We validated surface expression of IL1RAP in A6L cell line using flow cytometry and then demonstrated that two independent siRNAs were able to knockdown IL1RAP expression in this cell line (Fig. 1K). Functionally, siRNA mediated knockdown of IL1RAP led to reduced viability in A6L PDAC cells (Fig. 1L). siRNA mediated knockdown of IL1RAP also led to reduced colony growth from single cells and also led to decreased invasiveness measured by matrigel assays (Fig. 1M,N). siRNA mediated knockdown of IL1RAP also led to a significant G0/G1 cell cycle arrest (Fig. 2A,B). Transcriptomic analysis on A6L PDAC cells with and without siRNA mediated knockdown of IL1RAP demonstrated a reduction in cell cycle progression genes and pathways that included CDK1 and Topoisomerase 2a (Fig. 2C, Additional file 1: Tables S1, S2). Immunoblotting confirmed downregulation of these genes and also showed reduced levels of proliferative phospho/activated ERK in cells with IL1RAP knockdown (Fig. 2D,E).

**Fig. 2:**
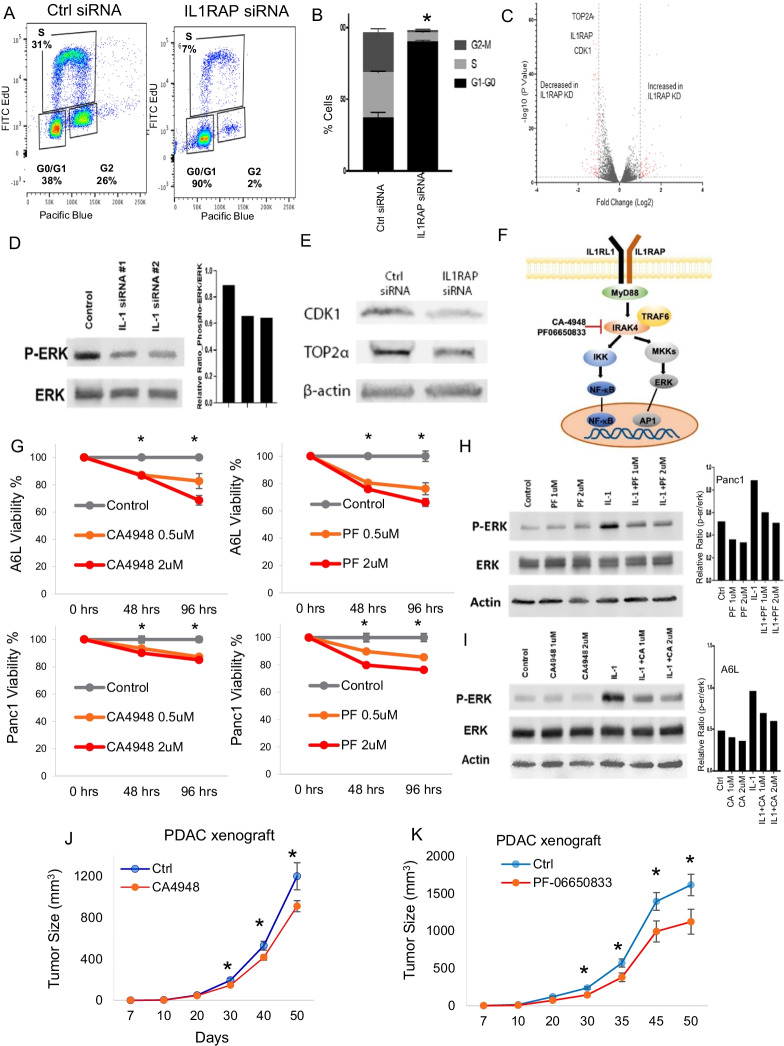
IL1RAP knockdown and IRAK4 inhibition reduce pancreatic cancer cell growth. **A**, **B**: siRNA mediated knockdown of IL1RAP leads to Go/G1 cell cycle arrest in A6L PDAC cells. Representative flow plots are shown. Means ± stdev, *N* = 10 (**B**). Ttest *P* Val < 0.01. **C**: RNAseq done on A6L PDAC cells with and without siRNA mediated knockdown of IL1RAP show reduction in numerous genes. **D**, **E**: Immunoblotting shows reduced levels of proliferative phosphor/activated erk MAPK kinase (**D**) and CDK1 and Topoisomerase 2a (**E**) in cells with IL1RAP knockdown. **F**: IRAK4 is a targetable kinase downstream of IL1RAP activation. CA-4948 and PF06650833 are clinically active small molecule specific inhibitors of IRAK4. **G**: Pharmacologic inhibition of IRAK4 with CA4948 and PF06650833 leads to reduced viability in Panc1 and A6L PDAC cells. Means ± stdev, *N* = 3, Ttest *P Val < 0.05. **H**, **I**: Immunblotting shows reduced levels of proliferative phospho/activated erk MAPK kinase after IRAK4 inhibition in A6L PDAC cells. **J**, **K**: PDAC xenografts were established with subcutaneous A6L implantation in NSG mice. Treatment with 50 mg/kg/d of CA4948 (**J**) and PF06650833 (**K**) with oral gavage was initiated and tumor sizes were measured. Significant reduction in tumor volumes was observed with inhibition of IRAK4 in vivo. (Means ± s.e.m, *N* = 28 for CA4948; *N* = 10 for PF06650833). *P Val < 0.05)

IRAK4 is a targetable kinase downstream of IL1RAP activation and can be specifically inhibited by small molecular inhibitors CA-4948 and PF06650833 [6, 7] (Fig. 2F). We observed that pharmacologic inhibition of IRAK4 with CA4948 and PF06650833 led to reduced viability in A6L and Panc-1 cells (Fig. 2G). Immunoblotting showed reduced levels of proliferative phospho/activated Erk after IRAK4 inhibition in Panc-1 and A6L cells (Fig. 2H,I). Finally, subcutaneous PDAC xenografts were established using A6L cells in immune deficient NSG mice. Treatment with 50 mg/kg/d of CA4948 (Fig. 2J) and PF06650833 (Fig. 2K) with oral gavage was initiated and tumor sizes were measured serially. Significant reduction in tumor volumes was observed with inhibition of IRAK4 in vivo demonstrating the potential of therapeutically targeting this pathway (Fig. 2J,K) (Additional file 2: Methods).

The tumor microenvironment plays a critical role in promoting the growth and invasion of pancreatic cancer cells [8]. Inflammatory signaling including IL1 and other cytokines has been implicated in pancreatic carcinogenesis in multiple studies [9–11]. In this report, we demonstrate IL1RAP as a novel, expressed surface marker in pancreatic ductal adenocarcinoma and show that knockdown or pharmacologic inhibition of IL1RAP pathways can result in growth inhibition in pancreatic cancer cells both in vitro and in vivo .

## Supplementary Information

**Additional file 1.** Cell cycle genes dysregulated after IL1RAP knockdown.

**Additional file 2.** Methods.

## Data Availability

RNAseq information has been submitted to GEO (ID pending).

## Abbreviations

IL1RAP: IL1 receptor accessory protein
PDAC: Pancreatic ductal adenocarcinoma
IRAK4: Interleukin-1 receptor-associated kinase 4
IPMN: Intraductal papillary mucinous neoplasm
