# Correction: Innate immune mediator, Interleukin-1 receptor accessory protein (IL1RAP), is expressed and pro-tumorigenic in pancreatic cancer

**DOI:** 10.1186/s13045-022-01321-4

**Published:** 2022-07-26

**Authors:** Yang Zhang, Xiaoyi Chen, Huamin Wang, Shanisha Gordon-Mitchell, Srabani Sahu, Tushar D. Bhagat, Gaurav Choudhary, Srinivas Aluri, Kith Pradhan, Plabani Sahu, Milagros Carbajal, Hui Zhang, Beamon Agarwal, Aditi Shastri, Robert Martell, Daniel Starczynowski, Ulrich Steidl, Anirban Maitra, Amit Verma

**Affiliations:** 1 Albert Einstein College of Medicine, Montefiore Medical Center, 1300 Morris Park Avenue, Bronx, NY 10461 USA; 2 Departments of Anatomical Pathology, University of Texas MD Anderson Cancer Center, Houston, TX USA; 3 Translational Molecular Pathology, University of Texas MD Anderson Cancer Center, Houston, TX USA; 4 GenomeRxUs LLC, Secane, PA USA; 5 Curis Inc, Lexington, MA USA; 6 Cincinnati Children’s Hospital, Cincinnati, OH USA

## Correction to: Journal of Hematology & Oncology (2022) 15:70 10.1186/s13045-022-01286-4

The original article erroneously omitted the following Conflict of Interest statement:

Dr. Maitra receives royalties from Cosmos Wisdom Biotech for a license related to a pancreatic cancer early detection test. He is also listed as an inventor on a patent licensed to Thrive Earlier Detection Ltd (an Exact Sciences Company) and serves as a consultant for Freenome and Tezcat Biotechnology.

