# Supplementary material for: Innate immune mediator, Interleukin-1 receptor accessory protein (IL1RAP), is expressed and pro-tumorigenic in pancreatic cancer
Source: J Hematol Oncol. 2022 May 23;15:70. doi: 10.1186/s13045-022-01286-4 (PMC9128118; doi:10.1186/s13045-022-01286-4)
Supplement: Supplementary file 1 — Additional file 1. Cell cycle genes dysregulated after IL1RAP knockdown. [file 13045_2022_1286_MOESM1_ESM.pptx]

## Slide 1
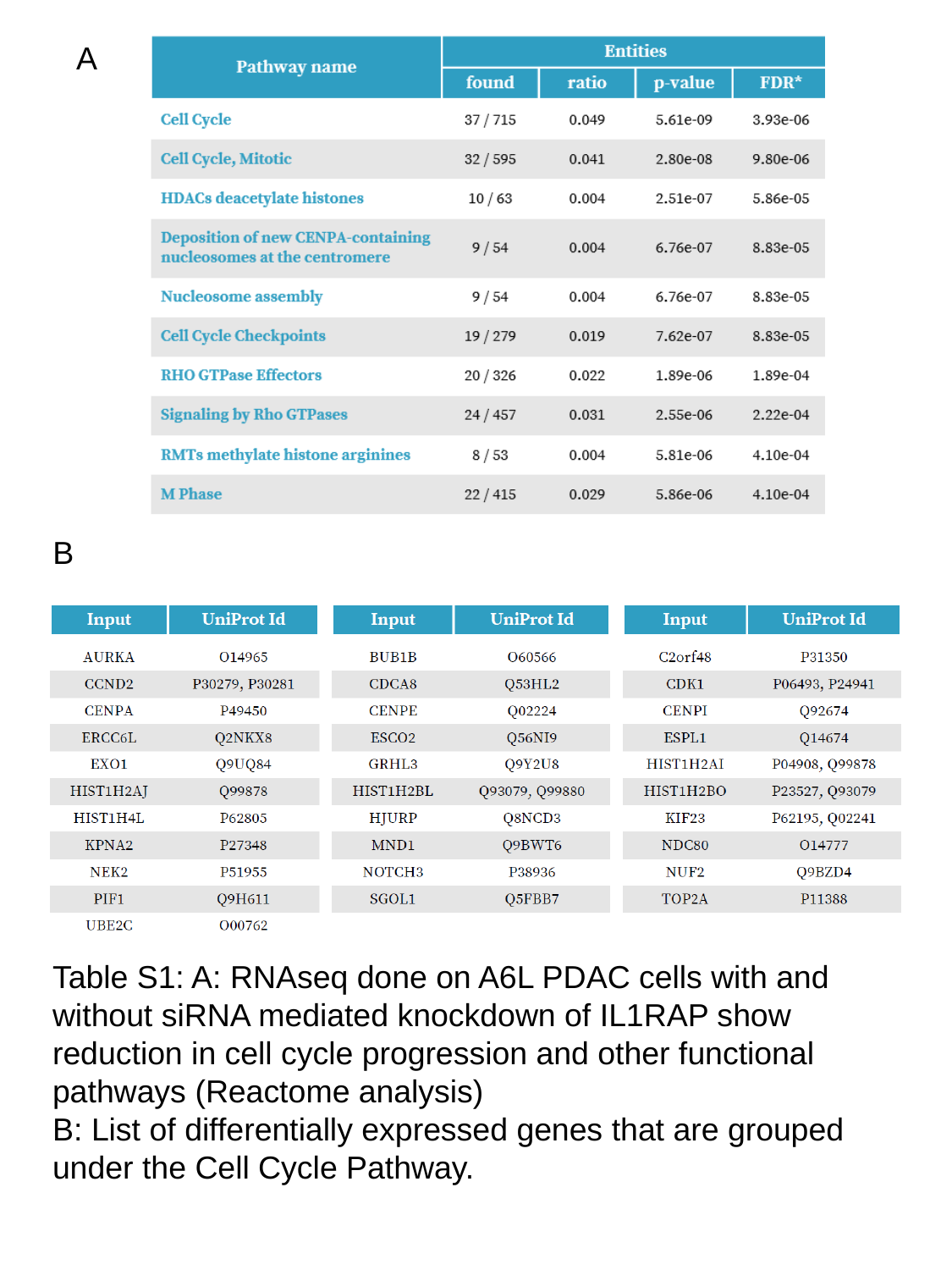

A
B
Table S1: A: RNAseq done on A6L PDAC cells with and without siRNA mediated knockdown of IL1RAP show reduction in cell cycle progression and other functional pathways (Reactome analysis)
B: List of differentially expressed genes that are grouped under the Cell Cycle Pathway.

## Slide 2
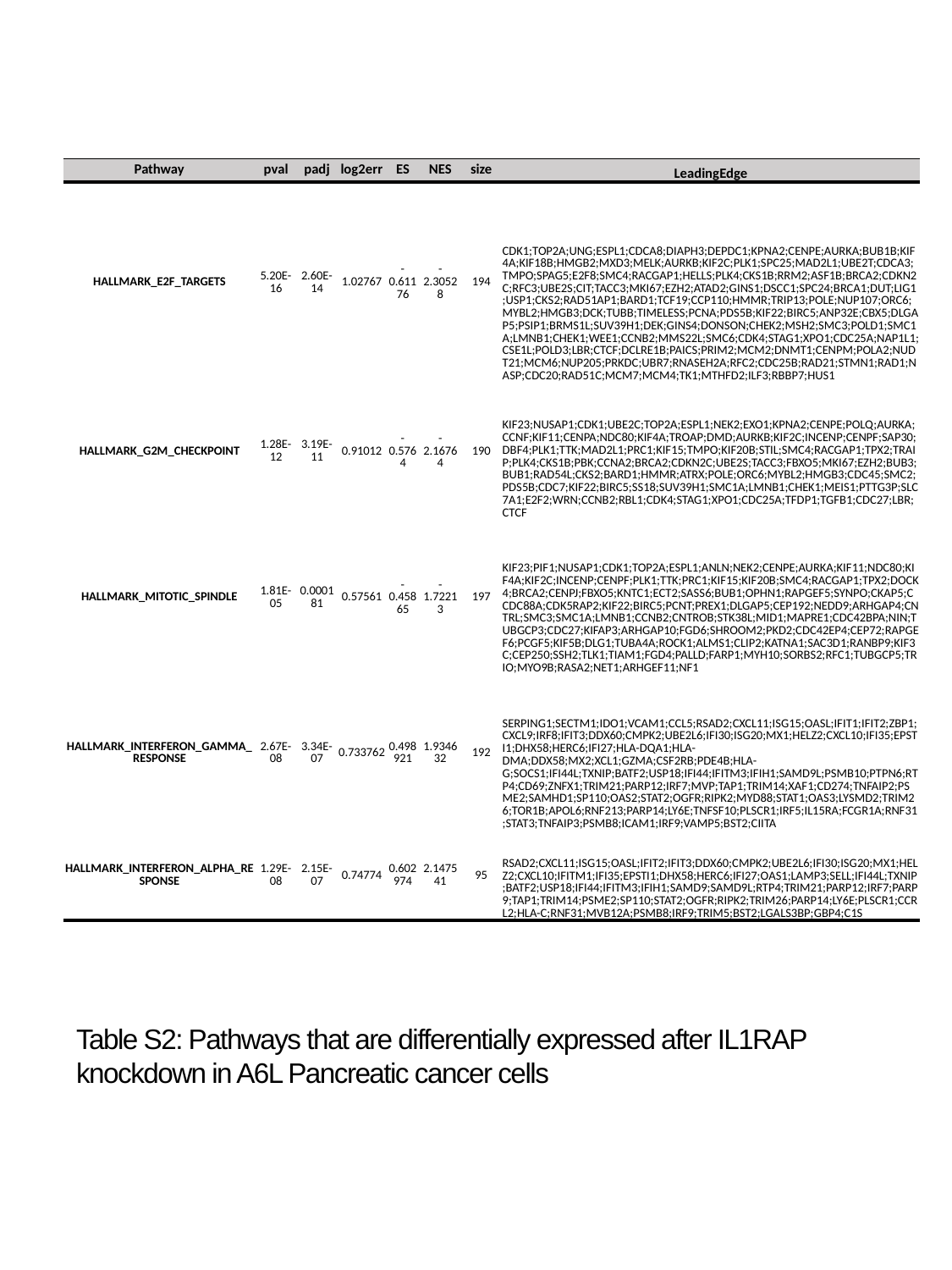

| Pathway | pval | padj | log2err | ES | NES | size | LeadingEdge |
| --- | --- | --- | --- | --- | --- | --- | --- |
| HALLMARK\_E2F\_TARGETS | 5.20E-16 | 2.60E-14 | 1.02767 | -0.61176 | -2.30528 | 194 | CDK1;TOP2A;UNG;ESPL1;CDCA8;DIAPH3;DEPDC1;KPNA2;CENPE;AURKA;BUB1B;KIF4A;KIF18B;HMGB2;MXD3;MELK;AURKB;KIF2C;PLK1;SPC25;MAD2L1;UBE2T;CDCA3;TMPO;SPAG5;E2F8;SMC4;RACGAP1;HELLS;PLK4;CKS1B;RRM2;ASF1B;BRCA2;CDKN2C;RFC3;UBE2S;CIT;TACC3;MKI67;EZH2;ATAD2;GINS1;DSCC1;SPC24;BRCA1;DUT;LIG1;USP1;CKS2;RAD51AP1;BARD1;TCF19;CCP110;HMMR;TRIP13;POLE;NUP107;ORC6;MYBL2;HMGB3;DCK;TUBB;TIMELESS;PCNA;PDS5B;KIF22;BIRC5;ANP32E;CBX5;DLGAP5;PSIP1;BRMS1L;SUV39H1;DEK;GINS4;DONSON;CHEK2;MSH2;SMC3;POLD1;SMC1A;LMNB1;CHEK1;WEE1;CCNB2;MMS22L;SMC6;CDK4;STAG1;XPO1;CDC25A;NAP1L1;CSE1L;POLD3;LBR;CTCF;DCLRE1B;PAICS;PRIM2;MCM2;DNMT1;CENPM;POLA2;NUDT21;MCM6;NUP205;PRKDC;UBR7;RNASEH2A;RFC2;CDC25B;RAD21;STMN1;RAD1;NASP;CDC20;RAD51C;MCM7;MCM4;TK1;MTHFD2;ILF3;RBBP7;HUS1 |
| HALLMARK\_G2M\_CHECKPOINT | 1.28E-12 | 3.19E-11 | 0.91012 | -0.5764 | -2.16764 | 190 | KIF23;NUSAP1;CDK1;UBE2C;TOP2A;ESPL1;NEK2;EXO1;KPNA2;CENPE;POLQ;AURKA;CCNF;KIF11;CENPA;NDC80;KIF4A;TROAP;DMD;AURKB;KIF2C;INCENP;CENPF;SAP30;DBF4;PLK1;TTK;MAD2L1;PRC1;KIF15;TMPO;KIF20B;STIL;SMC4;RACGAP1;TPX2;TRAIP;PLK4;CKS1B;PBK;CCNA2;BRCA2;CDKN2C;UBE2S;TACC3;FBXO5;MKI67;EZH2;BUB3;BUB1;RAD54L;CKS2;BARD1;HMMR;ATRX;POLE;ORC6;MYBL2;HMGB3;CDC45;SMC2;PDS5B;CDC7;KIF22;BIRC5;SS18;SUV39H1;SMC1A;LMNB1;CHEK1;MEIS1;PTTG3P;SLC7A1;E2F2;WRN;CCNB2;RBL1;CDK4;STAG1;XPO1;CDC25A;TFDP1;TGFB1;CDC27;LBR;CTCF |
| HALLMARK\_MITOTIC\_SPINDLE | 1.81E-05 | 0.000181 | 0.57561 | -0.45865 | -1.72213 | 197 | KIF23;PIF1;NUSAP1;CDK1;TOP2A;ESPL1;ANLN;NEK2;CENPE;AURKA;KIF11;NDC80;KIF4A;KIF2C;INCENP;CENPF;PLK1;TTK;PRC1;KIF15;KIF20B;SMC4;RACGAP1;TPX2;DOCK4;BRCA2;CENPJ;FBXO5;KNTC1;ECT2;SASS6;BUB1;OPHN1;RAPGEF5;SYNPO;CKAP5;CCDC88A;CDK5RAP2;KIF22;BIRC5;PCNT;PREX1;DLGAP5;CEP192;NEDD9;ARHGAP4;CNTRL;SMC3;SMC1A;LMNB1;CCNB2;CNTROB;STK38L;MID1;MAPRE1;CDC42BPA;NIN;TUBGCP3;CDC27;KIFAP3;ARHGAP10;FGD6;SHROOM2;PKD2;CDC42EP4;CEP72;RAPGEF6;PCGF5;KIF5B;DLG1;TUBA4A;ROCK1;ALMS1;CLIP2;KATNA1;SAC3D1;RANBP9;KIF3C;CEP250;SSH2;TLK1;TIAM1;FGD4;PALLD;FARP1;MYH10;SORBS2;RFC1;TUBGCP5;TRIO;MYO9B;RASA2;NET1;ARHGEF11;NF1 |
| HALLMARK\_INTERFERON\_GAMMA\_RESPONSE | 2.67E-08 | 3.34E-07 | 0.733762 | 0.498921 | 1.934632 | 192 | SERPING1;SECTM1;IDO1;VCAM1;CCL5;RSAD2;CXCL11;ISG15;OASL;IFIT1;IFIT2;ZBP1;CXCL9;IRF8;IFIT3;DDX60;CMPK2;UBE2L6;IFI30;ISG20;MX1;HELZ2;CXCL10;IFI35;EPSTI1;DHX58;HERC6;IFI27;HLA-DQA1;HLA-DMA;DDX58;MX2;XCL1;GZMA;CSF2RB;PDE4B;HLA-G;SOCS1;IFI44L;TXNIP;BATF2;USP18;IFI44;IFITM3;IFIH1;SAMD9L;PSMB10;PTPN6;RTP4;CD69;ZNFX1;TRIM21;PARP12;IRF7;MVP;TAP1;TRIM14;XAF1;CD274;TNFAIP2;PSME2;SAMHD1;SP110;OAS2;STAT2;OGFR;RIPK2;MYD88;STAT1;OAS3;LYSMD2;TRIM26;TOR1B;APOL6;RNF213;PARP14;LY6E;TNFSF10;PLSCR1;IRF5;IL15RA;FCGR1A;RNF31;STAT3;TNFAIP3;PSMB8;ICAM1;IRF9;VAMP5;BST2;CIITA |
| HALLMARK\_INTERFERON\_ALPHA\_RESPONSE | 1.29E-08 | 2.15E-07 | 0.74774 | 0.602974 | 2.147541 | 95 | RSAD2;CXCL11;ISG15;OASL;IFIT2;IFIT3;DDX60;CMPK2;UBE2L6;IFI30;ISG20;MX1;HELZ2;CXCL10;IFITM1;IFI35;EPSTI1;DHX58;HERC6;IFI27;OAS1;LAMP3;SELL;IFI44L;TXNIP;BATF2;USP18;IFI44;IFITM3;IFIH1;SAMD9;SAMD9L;RTP4;TRIM21;PARP12;IRF7;PARP9;TAP1;TRIM14;PSME2;SP110;STAT2;OGFR;RIPK2;TRIM26;PARP14;LY6E;PLSCR1;CCRL2;HLA-C;RNF31;MVB12A;PSMB8;IRF9;TRIM5;BST2;LGALS3BP;GBP4;C1S |
# Table S2: Pathways that are differentially expressed after IL1RAP knockdown in A6L Pancreatic cancer cells
