# Supplementary material for: Innate immune mediator, Interleukin-1 receptor accessory protein (IL1RAP), is expressed and pro-tumorigenic in pancreatic cancer
Source: J Hematol Oncol. 2022 May 23;15:70. doi: 10.1186/s13045-022-01286-4 (PMC9128118; doi:10.1186/s13045-022-01286-4)
Supplement: Supplementary file 2 — Additional file 2. Methods. [file 13045_2022_1286_MOESM2_ESM.docx]

**Additional file 2: Methods**

**Cell lines and reagents:** The human pancreatic ductal adenocarcinoma cell line A6L (Pa02C) ([12](#_ENREF_12)), was maintained in DMEM complete media supplemented with 10% FBS and 1% penicillin-streptomycin. IRAK4 inhibitor CA-4948 was obtained from Curis. PF06650833 was obtained from Selleck Chemical.

**Transfection of small interfering RNA (siRNA):** PDAC cell lines cells were seeded and allowed to adhere overnight in 6-well culture plates at a density of 2×10^5^ cells/well. Following overnight incubation, cells were transfected with 50nmol/L siRNA targeting IL1RAP (Dharmacon Technologies, Thermo Fisher Scientific, Lafayette, CO) or non-targeting control siRNA (Dharmacon Technologies) using DharmaFECT 4 transfection reagent. At 24h post-transfection, cells were plated for co-culture invasion assays.

**Transwell Coculture Invasion Assay:** 8µm pore size inserts were coated with 100µL Matrigel (1:40 Matrigel: PBS solution) (BD Biosciences, San Jose, CA) and allowed to solidify in a notched 24-well culture plate overnight. The media was then replaced with DMEM containing 1% FBS and A6L (Pa02C) cells were suspended in DMEM containing 0.5% FBS and seeded at 5×10^4^/well in the top (notched insert) chamber. Following 48hr incubation, the assay was terminated and cells migrating to the underside of the insert were fixed in ethanol and stained with 0.25% crystal violet solution. Each condition was performed in triplicate.

**Immunohistochemistry:** Tissue microarrays (TMAs) were generated using formalin-fixed, paraffin-embedded tissue samples. The mouse tissue samples and TMAs were sectioned in 5 µm thickness using a microtome. The slides were incubated at 60 ◦C for an hour to deparaffinize the tissue, followed by dehydrating them through gradients of ethanol (70, 80, 90 and 100%) and 100% xylene. The samples were then treated with antigen unmasking solution (Dako pharma) followed by permeabilization with 0.3% H2O2 and blocked using blocking buffer (5% donkey serum and 2% BSA). Samples were then incubated overnight in the primary antibody prepared in the blocking buffer followed an incubation with appropriate HRP conjugated secondary antibody. Color development was achieved by treating the samples with diaminobenzidine (DAB) and counterstaining performed using harris hematoxylene (Dako pharma). The samples were then passaged through alcohol grades and xylene to dehydrate them, mounted using permount solution (fisher scientific) and allowed to dry overnight. The staining results were graded into 1+, 2+ and 3+ based on the staining intensity in tumor cells.

**Xenografts:** A6L PDAC cells were xenografted subcutaneously in NSG mice. After tumors were established, the mice were treated with placebo or IRAK4 inhibitors (CA4948 or PF06650833) at 50 mg/kg dose by oral gavage daily on a 5days/week regimen. Tumor measurements were taken at periodic intervals.
